# AI semantics for biomedical data integration

**DOI:** 10.64898/2026.08.03.742514

**Published:** 2026-08-07

**Authors:** James McLaughlin, Aleix Puig-Barbe, Arwa Ibrahim, Diego Pava, Zoe May Pendlington, Nicolas Matentzoglu, Elliot Sollis, Amy Foreman, Robert Wilson, Federico Lopez Gomez, Laura Harris, Yeyejide Adeleye, Satwant Kaur, Birgit Meldal, Damian Smedley, Helen Parkinson

## Abstract

Researchers increasingly need to explore hypotheses that span multimodal data across different scales, organisms, and domains. In practice, this requires connecting knowledge across fragmented databases with incompatible APIs and heterogeneous annotation practices. Large language model (LLM) agents can automate this data integration process, but grounding LLM agent outputs in scientifically correct sources of truth remains a significant challenge.

Here we describe our deployment of a novel AI semantics workflow using LLM agents to enable scalable data integration, grounded in biological knowledge in the form of ontologies. Our workflow comprises (1) a multi-agent system curating scientific knowledge across ontologies using the Ontology Lookup Service (OLS) as grounding; (2) an LLM embedding service to enable interoperability between scientific databases by mapping ontology terms; and (3) GrEBI, a knowledge graph and Model Context Protocol (MCP) server enabling LLM agents to conduct cross-cutting, multi-omic biomedical queries.

## Full Text Availability

The license terms selected by the author(s) for this preprint version do not permit archiving in PMC. The full text is available from the preprint server.

